# Bisdioxycalamenene: A Bis-Sesquiterpene from the Soft Coral *Rhytisma fulvum fulvum*

**DOI:** 10.3390/md14020041

**Published:** 2016-02-19

**Authors:** Yuval J. Trifman, Maurice Aknin, Anne Gauvin-Bialecki, Yehuda Benayahu, Shmuel Carmeli, Yoel Kashman

**Affiliations:** 1 School of Chemistry, Raymond and Beverly Sackler Faculty of Exact Sciences, Tel Aviv University, Tel Aviv 69978, Israel; yuvaltri@mail.tau.ac.il (Y.J.T.); carmeli@post.tau.ac.il (S.C.); 2 Laboratory of Chemistry of Natural Substances and Food Sciences, Faculty of Science and Technology, University of Reunion Island, 15 Avenue René Cassin, CS 92003, 97744 Saint-Denis Cedex 9, La Reunion, France; maurice.aknin@univ-reunion.fr (M.A.); anne.bialecki@univ-reunion.fr (A.G.-B.); 3 Department of Zoology, George S. Wise Faculty of Life Sciences, Tel Aviv University, Tel Aviv 69978, Israel; yehudab@tauex.tau.ac.il

**Keywords:** *Rhytisma fulvum fulvum*, soft coral, bisdioxycalamenene, bis-sesquiterpene

## Abstract

A dichloromethane extract of the soft coral *Rhytisma fulvum fulvum* collected in Madagascar afforded a novel compound possessing an unprecedented pentacyclic skeleton, bisdioxycalamenene (**1**), as well as seven known sesquiterpenes. The structures of the compounds were elucidated using 1D and 2D NMR techniques, as well as high-resolution mass spectrometry. The absolute configuration of **1** was determined using X-ray diffraction analysis and anomalous dispersion effects. The structure elucidation and a possible biogenesis of the compound are discussed.

## 1. Introduction

The soft coral *Rhytisma fulvum fulvum* (Forskål) (*R. fulvum fulvum*) (previously *Parerythropodium*), family Alcyoniidae, was originally described from the Red Sea [1,2]. Its present zoogeographical distribution extends south to the reefs of Madagascar and eastward to Indonesia [3,4]. The Genera *Rhytisma* is a rich source of sesquiterpenoids and norsesquiterpenoids containing a variety of skeletons as well as sterols [5,6,7,8,9]. Dozens of compounds have thus far been reported with different ring systems [5,6,7,8,9]. In previous research, our group compared the yellow and gray morphs that inhabit the reefs of the gulf of Eilat and found differences in the compounds they produce [6]. *Inter alia*, we isolated a volatile yellow pigment, fulfulvene, from the yellow morph. This pigment is responsible for the soft coral’s yellow color [6]. The pigment and other volatile compounds, mainly sesquiterpenes, are lost during freeze drying and/or evaporation [6].

**Figure 1:**
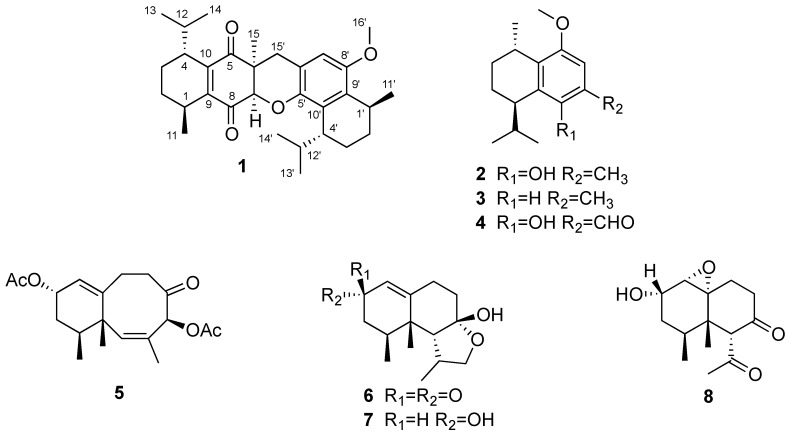
*Rhytisma fulvum fulvum* metabolites isolated in the current study.

The present study describes the isolation and structure elucidation of a novel bis-sesquiterpene designated bisdioxycalamenene (**1**) and of seven known sesquiterpenes, 5-hydroxy-8-methoxycalamenene (**2**) [10], 8-methoxycalamenene (**3**) [10], 5-hydroxy-8-methoxycalamenene-15-al (**4**) [10], (1*S*,4*S*,10*S*,12*S*,*Z*)-1,3,12-trimethyl-5-oxo-1,4,5,6,7,10,11,12-octahydrobenzo[8]-annulene-4,10-diyldi-acetate (**5**) [11], 2-oxolemnacarnol (**6**) [5], lemnacarnol (**7**) [5], and 6α-acetyl-4β,5β-dimethyl-1(10)-α-epoxy-2α-hydroxy-7-oxodecalin (**8**) [12], from a yellow morph specimen of *Rhytisma fulvum fulvum* collected on December 2012 at Banc du Castor, Mitsios Archipelago, Madagascar (Figure 1). Bisdioxycalamenene (**1**) is a bis-sesquiterpene possessing an unprecedented pentacyclic skeleton that is probably derived from dimerization of 5-hydroxy-8-methoxycalamenene (**2**) [10]. 

## 2. Results and Discussion

Bisdioxycalamenene (**1**) was isolated from the crude extract using solvent partition, followed by separations on Sephadex LH-20 and Silica gel-H. The positive atmospheric pressure photoionization high-resolution mass spectrometry (APPI HRMS) of **1** exhibited a molecular ion [M]^+^ at *m*/*z* 478.3080 consistent with the molecular formula C_31_H_42_O_4_ and eleven double bond equivalents. The structure elucidation of **1** was based on its mass-spectrometric and NMR data (Table 1, CDCl_3_). The ^1^H NMR exhibited two singlet signals in the lower and mid-field of the spectrum (δ_H_ 6.36 and 4.39), a methoxyl (δ_H_ 3.74), six protons resonating between 3.40 and 2.50 ppm, ten sp^3^ protons resonating between 2.00 and 1.40 ppm, a singlet methyl (δ_H_ 1.33) and six doublet methyl signals resonating between 1.10 and 0.75 ppm. The ^13^C NMR presented two ketone carbonyls (δ_C_ 199.4 s and 195.3 s), eight sp^2^ carbon atoms (δ_C_ 151.7, 150.3, 147.3, 144.0, 130.6, 129.1 and 115.4, all quaternary carbons, and 107.5, CH), a oxymethine carbon (δ_C_ 83.9, CH), and a methoxyl (δ_C_ 55.2, CH_3_). In the high field, one quaternary sp^3^ carbon (δ_C_ 48.0), six methine carbons (δ_C_ 37.0, 36.9, 32.3, 31.1, 27.2 and 26.5), four methylene carbons (δ_C_ 25.6, 25.5, 19.5 and 18.5) and seven methyl carbons (δ_C_ 21.7, 21.4, 21.3, 20.2, 21.9, 20.0 and 21.8) were observed. The above proton and carbon signals counted to 31 carbons and 42 protons, agreeing with the molecular formula of **1**.

**Figure 2:**
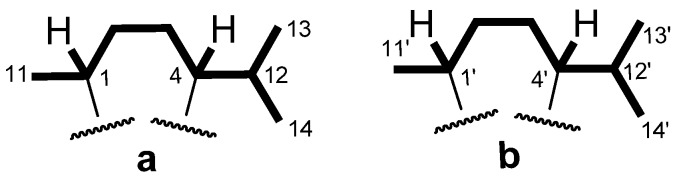
COSY (bold line) correlations of **1**.

The C-H correlations from a heteronuclear single quantum coherence (HSQC) experiment established the one bond connectivity between the carbons and protons (Table 1). The H-H correlations from a correlation spectroscopy (COSY) experiment furnished two similar segments; **a** and **b**, shown in Figure 2, leaving an additional isolated strong coupled methylene group (δ_H_ 3.35 and 2.53 ppm) and four isolated proton signals (δ_H_ 6.36, s, 1H; 4.39, s, 1H; 3.74, s, 3H; 1.33, s, 3H) uncorrelated. Segments **a** and **b** are similar each other and both to the aliphatic portion of the calamenene sesquiterpenes (**2**–**4**) [10].

**Figure 3:**
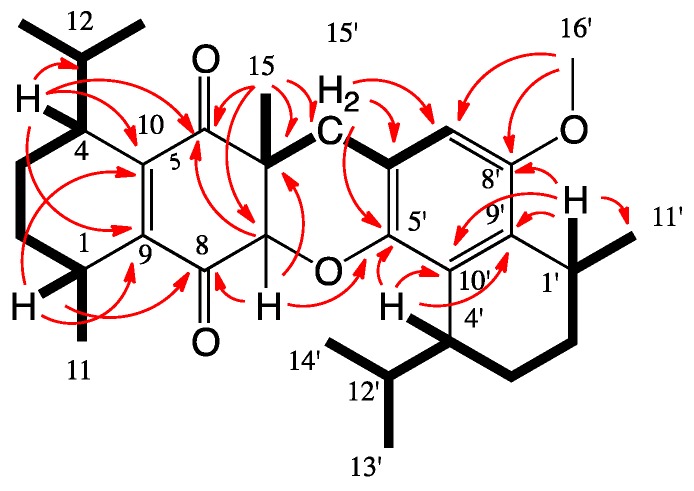
COSY (bold line) and key HMBC correlations (arrows) of **1**.

Using long-range C-H correlations from a heteronuclear multiple bond correlation (HMBC) experiment enabled the connection of all the proton and carbon signals to the final gross planar structure of **1** (Figure 3). H-1 and -4 exhibited correlations with C-8 and -5, respectively, and both with C-9 and -10, extending fragment **a** to a 1-isopropyl-4-methyl-2-cyclohexene moiety substituted at positions 2 and 3 by two ketone groups. Oxymethine-7 presented HMBC correlations with C-5, -6, -8 and -9, while methyl-15-protons exhibited correlations with C-5, -6, and -7, closing a second six-membered ring. H-1′ and -4′ demonstrated a similar pattern of correlations (Figure 3), extending fragment **b** to a 1,4-disubstituted cyclohexene ring. H-7′ exhibited HMBC correlations with C-1′, -5′, -6′, -8′, -9′ and -15′, while methylene-15′-protons presented correlations with C-5′, -6′, -7′ and -8′, and the methoxy-protons exhibited correlations with C-8′, supporting a substituted 15-methylene-8-methoxy-5-oxy-calamenene substructure. The HMBC correlations of H-7 with C-5′ and -15′, of methyl-15-protons with C-15′ and of both protons of methylene-15′ with C-5, -6 and -7 suggested the connection of C-6 with C-15′ and C-7 through the oxygen to C-5′, establishing the planar structure of **1**.

The relative *trans* relationships of the 1,4- and 1′,4′-substituents of the two cyclohexene rings were suggested when comparing the chemical shifts of the corresponding protons and carbons with those of the known calamenenes (**2**–**4**). The *cis* 6–7 junction was established based on the NOE between H-7 and methyl-15. The complete structure including absolute configuration was confirmed by the X-ray diffraction analysis.

**Figure 4:**
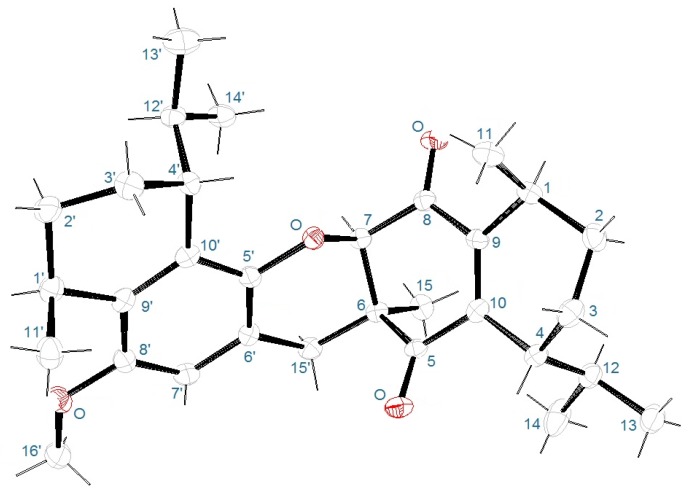
Oak Ridge Thermal-Ellipsoid Plot Program (ORTEP) presentation of **1**.

Crystallization of bisdioxycalamenene (**1**) from CHCl_3_/MeOH solution furnished suitable crystals for an X-ray diffraction analysis. Bisdioxycalamenene (**1**) comprises of a pentacyclic structure derived from coupling of two sesquiterpenes. The asymmetric unit contains assemblages of two molecules of **1**, thus in one monoclinic cell there are four molecules of **1**. Anomalous dispersion effects and relation of the two enantiomeric forms of the structural model to the diffracted intensities established the absolute configuration. The preferred fit is indicated by the Flack and Parsons parameter (Flack parameter, x = 0.01 (9)) [13]. The X-ray diffraction analysis established the complete structure of **1** including the absolute configuration of the six chiral centers (1*S*,1′*S*,4*R*,4′*R*,6*S*,7*R*) shown in Figure 4.

**Figure 5:**
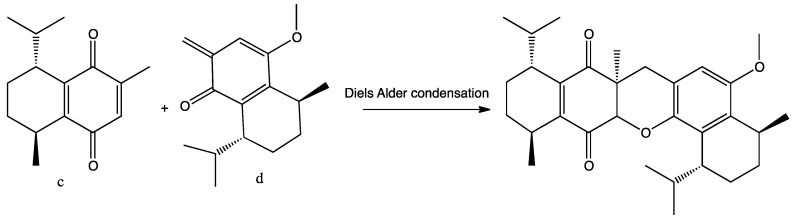
A possible biogenetic route for **1**.

To the best of our knowledge, there are no earlier reports of such a skeleton. A possible biogenetic route to **1** is a hetero Diels Alder condensation of the *para*-quinone **c** with the *ortho*-quinonemethide, **d** [14,15] (Figure 5).

Compound **1** was evaluated for lethal toxicity in a brine shrimp toxicity assay (*Artemia salina*) [16] and displayed mild toxicity (LD_50_ 15 μg/mL). Compound **1** was assayed for antibacterial activity against *Pseudomonas aeruginosa* and *Escherichia coli* and found to be inactive at 10 μg/mL.

## 3. Experimental Section

### 3.1. General Experimental Procedures

Optical rotations were determined on a JASCO P-1010 polarimeter. UV spectra were recorded on an Agilent 8453 spectrophotometer. NMR spectra were recorded on a Bruker DMX-500 spectrometer at 500.13 MHz for ^1^H and 125.76 MHz for ^13^C and a Bruker Avance 400 spectrometer at 400.13 MHz for ^1^H and 100.62 MHz for ^13^C. DEPT, COSY-45, gTOCSY (mixing time 60 ms), gROESY (spinlock pulse 0.2 s), gHSQC, and gHMBC spectra were recorded using standard Bruker pulse sequences. Mass spectra were recorded on a Synapt High Definition Mass Spectrometry (Waters Inc., Milford, MA, USA) instrument. For the GC MS analysis, separation of the crude samples was performed on an Agilent-7890-GC equipped with Agilent-5977A-MSD with an HP-5MS UI column (30 meter × 0.25 mm × 0.25 μm). The X-ray diffraction patterns were obtained with CuKα radiation from an Imus microsource, on an ApexDuo Bruker-AXS diffractometer.

### 3.2. Biological Material

A sample of the soft coral *R fulvum fulvum* (yellow morph, Phylum Cnideria, Class Anthozoa, Order Alcyonacea, Family Alcyoniidae) was collected in December 2012 at a depth of 15 m at Banc du Castor, Mitsios Archipelago, Madagascar. It was identified by Professor Yehuda Benayahu. A voucher specimen (MAD12-IM052) was deposited at the Laboratoire de Chimie des Substances Naturelles et des Sciences des Aliments (LCSNSA) at the University of Reunion Island, France. The soft coral sample was frozen immediately after collection and kept at −20 °C until processed.

### 3.3. Isolation Procedure

The wet sample (0.3 kg wet weight) of the soft coral was extracted with CH_2_Cl_2_–MeOH (1:1, 300 mL) at room temperature overnight. The aqueous phase was separated from the organic layer, which was dried and evaporated to afford an oily crude extract (9 g, 3% of dry weight). The crude organic extract was separated in 2 g portions using Kupchan solvent partition [17]. The petroleum ether fraction (970 mg) was chromatographed on a Sephadex LH-20 column (petroleum ether/CH_2_Cl_2_/MeOH, 2:1:1) followed by several separations on Silica gel 60 H (Merck) columns (VLC and gravitational columns), eluting with EtOAc-petroleum ether mixtures to afford bisdioxycalamenene (**1**) (eluted with 2% EtOAc in petroleum ether, 15 mg, 0.023% yield from wet sample) and seven known compounds: 5-hydroxy-8-methoxycalamenene (**2**) [10] (46.5 mg, 0.29% yield from wet sample), 8-methoxycalamenene (**3**) [10] (6 mg, 0.009% yield wet sample), 5-hydroxy-8-methoxycalamenene-15-al (**4**) [10] (0.0045% yield from wet sample), (1*S*,4*S*,10*S*,12*S*,*Z*)-1,3,12-trimethyl-5-oxo-1,4,5,6,7,10,11,12-octahydrobenzo[8]annulene-4,10-diyl diacetate (**5**) [11] (6 mg, 0.0045% yield from wet sample), 2-oxolemnacarnol (**6**) [5] (2 mg, 0.0024% yield from wet sample), lemnacarnol (**7**) [5] (4 mg, 0.003% yield from wet sample), and 6α-acetyl-4β,5β-dimethyl-1(10)-α-epoxy-2α-hydroxy-7-oxodecalin (**8**) [12] (3 mg, 0.0024% yield from wet sample).

Bisdioxycalamenene (**1**): yellowish crystals from CHCl_3_/MeOH; ${\left[\alpha \right]}_{D}^{23}$−175 (*c* 0.20, hexane); UV (Hexane) λ_max_ (log ε) 204 (4.07), 258 (3.44), 656 (1.70) nm; IR (ATR Diamond) ν_max_ 1684, 1613 cm^−1^; ^1^H and ^13^C NMR data, see Table 1; HRMS APPI+ exhibited a molecular ion [M]^+^ at *m*/*z* 478.3080 (calculated for C_31_H_42_O_4_, 478.3083).

### 3.4. X-Ray Crystallography

The structure of **1** was confirmed by single crystal X-ray diffraction analysis of a crystal obtained from a CHCl_3_/MeOH solution. The measurements were carried out on an ApexDuo (Bruker-AXS) diffractometer with CuKα radiation at low temperature in order to optimize the precision of the crystallographic determinations.

Crystal data: C_31_H_42_O_4_, M = 478.65, monoclinic space group P21, T = 110(2) K, DC = 1.217 g·cm^−3^, F(000) = 1040.0. Crystal structure approximate dimensions 0.106 × 0.265 × 0.493 mm^3^.

A total of 4174 frames were collected. The frames were integrated with the Bruker SAINT Software package using a narrow-frame algorithm. The integration of the data using a monoclinic unit cell yielded a total of 16,453 reflections to a maximum θ angle of 66.84° (0.84 Ǻ resolution), of which 7667 were independent (average redundancy 2.146, completeness = 97.0%, R_int_ = 3.22%, R_sig_ = 4.10%) and 7399 (96.50%) were greater than 2σ (F2). The final cell constants of: a = 9.5661(4) Ǻ, b = 10.5930(5) Ǻ, c = 25.8443(13) Ǻ, β = 93.725(3)°, volume = 2613.4(2) Ǻ3, are based upon the refinement of the XYZ-centroids of 132 reflections above 20 σ(I) with 19.19° < 2θ < 75.06°. Data were corrected for absorption effects using the multi-scan method (SADABS). CCDC number: 1414246.

### 3.5. Brine Shrimp Toxicity Assay

Bisdioxycalamenene (**1**) was evaluated for lethal toxicity in a brine shrimp (*Artemia salina*) toxicity assay [16], which displayed mild toxicity (LD_50_ > 10 μg/mL). The evaluation of brine shrimp toxicity (*Artemia salina*) was performed as previously reported [16].

## 4. Conclusions

The soft coral *R. fulvum fulvum* is widely distributed throughout the Indopacific [1,2,3,4] and present several color morphs. The genus *Rhytisma* is very rich in sesquiterpenes, norsesquiterpenes and other metabolites such as fulfulven that gives the yellow morph of *R. fulvum fulvum* its color [1,2,3,4,5,6,7,8,9]. In the present study, we have isolated, from a specimen collected in Madagascar, a sesquiterpene dimer of new pentacyclic skeleton, bisdioxycalamenene (**1**), along with seven previously described metabolites of *R. fulvum fulvum*. Although **1** did not present significant biological activity in the set of bioassays, we believe that there should be a good reason for its biosynthesis and for the investment of considerable amount of energy in it. Such purpose might be, for example, involvement in the reproduction process of soft coral as demonstrated for alkyl acetylenes produced by the hard coral *Montipora digitata* and the cembrane type diterpenoids in the soft coral *Lobophytum compactum* [18].

## Figures and Tables

**Table 1:** NMR Data of Bisdioxycalamenene (**1**) in CDCl_3_^a^.

Position	δ_C_, Type ^b^	δ_H_, Mult (*J* in Hz)	HMBC Correlations ^c^	COSY Correlations
1	27.2, CH	2.99, ddq (1.3, 6.8, 7.0)	2, 3, 5, 8, 9, 10, 11	2a, 11
2	25.5, CH_2_	1.84, m	10	1
		1.46, m		3a, 3b, 12
3	18.5, CH_2_	1.74, m	10	2b, 3b, 4, 12
		1.61, m	4, 10	2b, 3a, 4
4	36.9, CH	2.66, dt (2.3, 6.0)	2, 3, 5, 9, 10, 12, 14	3a, 3b, 12
5	199.4, qC			
6	48.0, qC			
7	83.9, CH	4.39, s	5, 6, 8, 9, 15, 5′, 15′	
8	195.3, qC			
9	150.3, qC			
10	147.3, qC			
11	20.1, CH_3_	1.10, d (7.0)	1, 2, 9, 10	1
12	31.1, CH	1.82, m	3, 4	2b, 3a, 4, 13, 14
13	21.3, CH_3_	0.87, d (7.0)	4, 12, 14	12
14	20.2, CH_3_	0.85, d (7.0)	4, 12	12
15	21.8, CH_3_	1.33, s	5, 6, 7, 6′, 15′	
1′	26.5, CH	3.09, dq (6.5,7.0)	2′, 3′, 8′, 9′, 10′, 11′	2′a, 11′
2′	25.6, CH2	1.93, m	10′	1′, 2′b, 3′
		1.41, m	4′, 9′	3′
3′	19.5, CH2	1.70, m	1′, 4′, 10′, 12′	2′a, 2′b, 4′
4′	37.0, CH	2.72, dt (2.3, 5.9)	2′, 5′, 9′, 10′, 12′, 14′	1′, 3′, 12′
5′	144.0, qC			
6′	115.4, qC			
7′	107.5, CH	6.36, s	5′, 8′, 9′, 15′	
8′	151.7, qC			
9′	130.6, qC			
10′	129.1, qC			
11′	21.9, CH3	1.07, d (7.0)	1′, 2′, 9′	1′
12′	32.3, CH	1.86, m	3′, 10′, 14′	4′, 13′, 14′
13′	21.7, CH3	0.86, d (7.0)	4′, 12′	12′
14′	21.4, CH3	0.77, d (7.0)	4′, 12′	12′
15′	33.1, CH2	3.35, d (16.5)	5, 6, 7, 8, 15, 5′, 6′, 7′, 8′, 9′	
		2.53, d (16.5)	5, 6, 7, 15, 5′, 6′, 7′, 8′, 9′, 10′	
16′	55.2, CH3	3.74, s	7′, 8′	

^a^ 500 MHz for ^1^H, 125 MHz for ^13^C; ^b^ Multiplicity and assignment from HSQC experiment; ^c^ HMBC correlations, optimized for 8 Hz, are from the proton(s) stated to the indicated carbon.

## Supplementary Materials

1D (^1^H, ^13^C) and 2D NMR (HSQC, HMBC, COSY) spectra and HR MS data of compound **1**, and photograph of the soft coral, are available.
