# Supplementary material for: Bisdioxycalamenene: A Bis-Sesquiterpene from the Soft Coral Rhytisma fulvum fulvum
Source: Mar Drugs. 2016 Feb 19;14(2):41. doi: 10.3390/md14020041 (PMC4771994; doi:10.3390/md14020041)
Supplement: Supplementary file 1 [file marinedrugs-14-00041-s001.pdf]

Supplementary Materials: Bisdioxycalamenene: A Bis-Sesquiterpene from the Soft Coral *Rhytisma fulvum fulvum*

Yuval J. Trifman, Maurice Aknin, Anne Gauvin-Bialecki, Yehuda Benayahu, Shmuel Carmeli and Yoel Kashman

Table of Contents

Figure S1. ^{1}H NMR of Bisdioxycalamenene (**1**) in $CDCl_3$	S2
Figure S2. ^{13}C NMR of Bisdioxycalamenene (**1**) in $CDCl_3$	S3
Figure S3. HSQC Spectrum of Bisdioxycalamenene (**1**) in $CDCl_3$	S4
Figure S4. HMBC Spectrum of Bisdioxycalamenene (**1**) in $CDCl_3$	S5
Figure S5. COSY Spectrum of Bisdioxycalamenene (**1**) in $CDCl_3$	S6
Figure S6. HRMS of Bisdioxycalamenene (**1**)	S7
Figure S7. Image of Rhytisma *fulvum fulvum*	S8

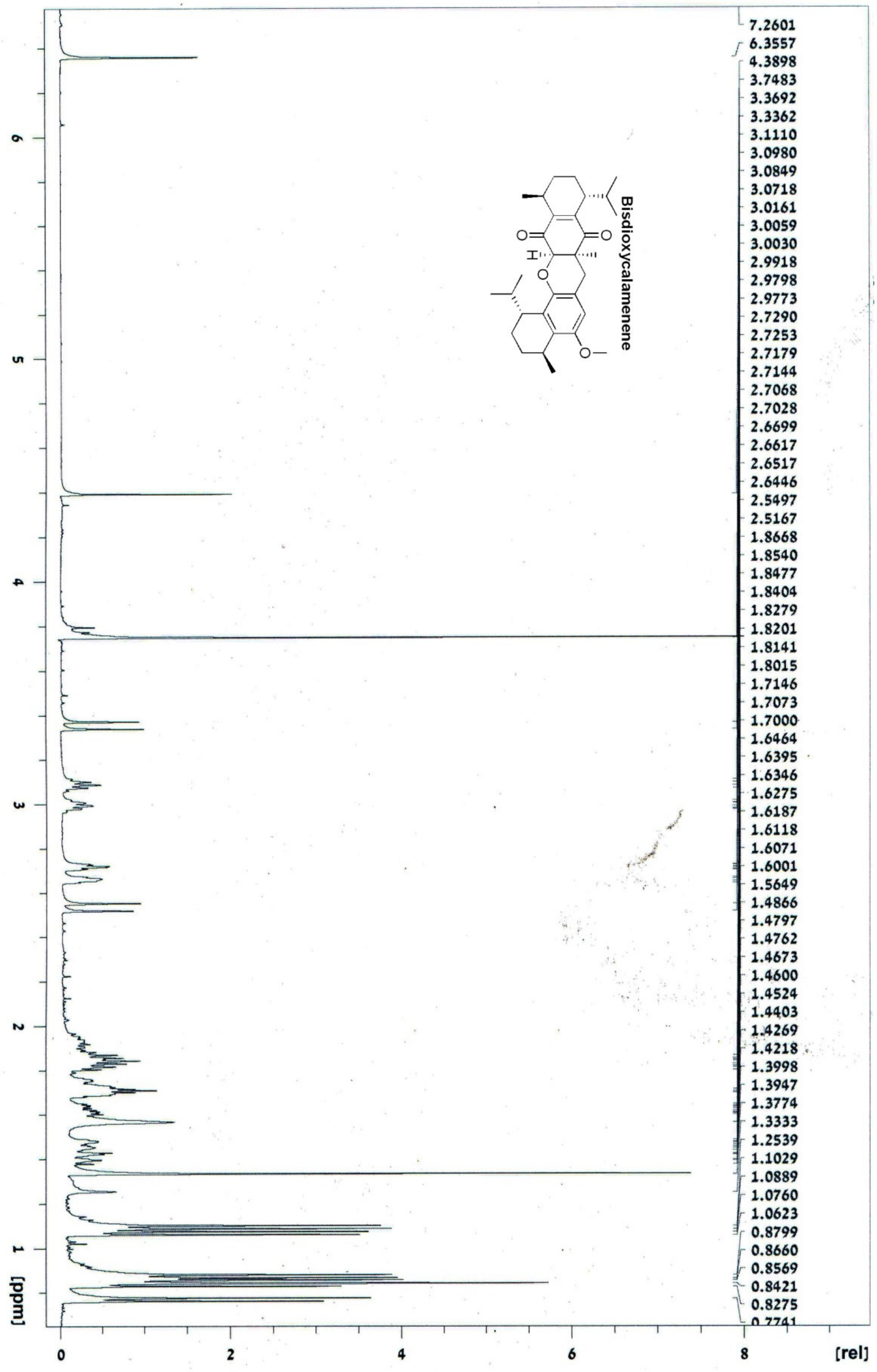

Figure S1. ^{1}H NMR of Bisdioxycalamenene (**1**) in $CDCl_3$.

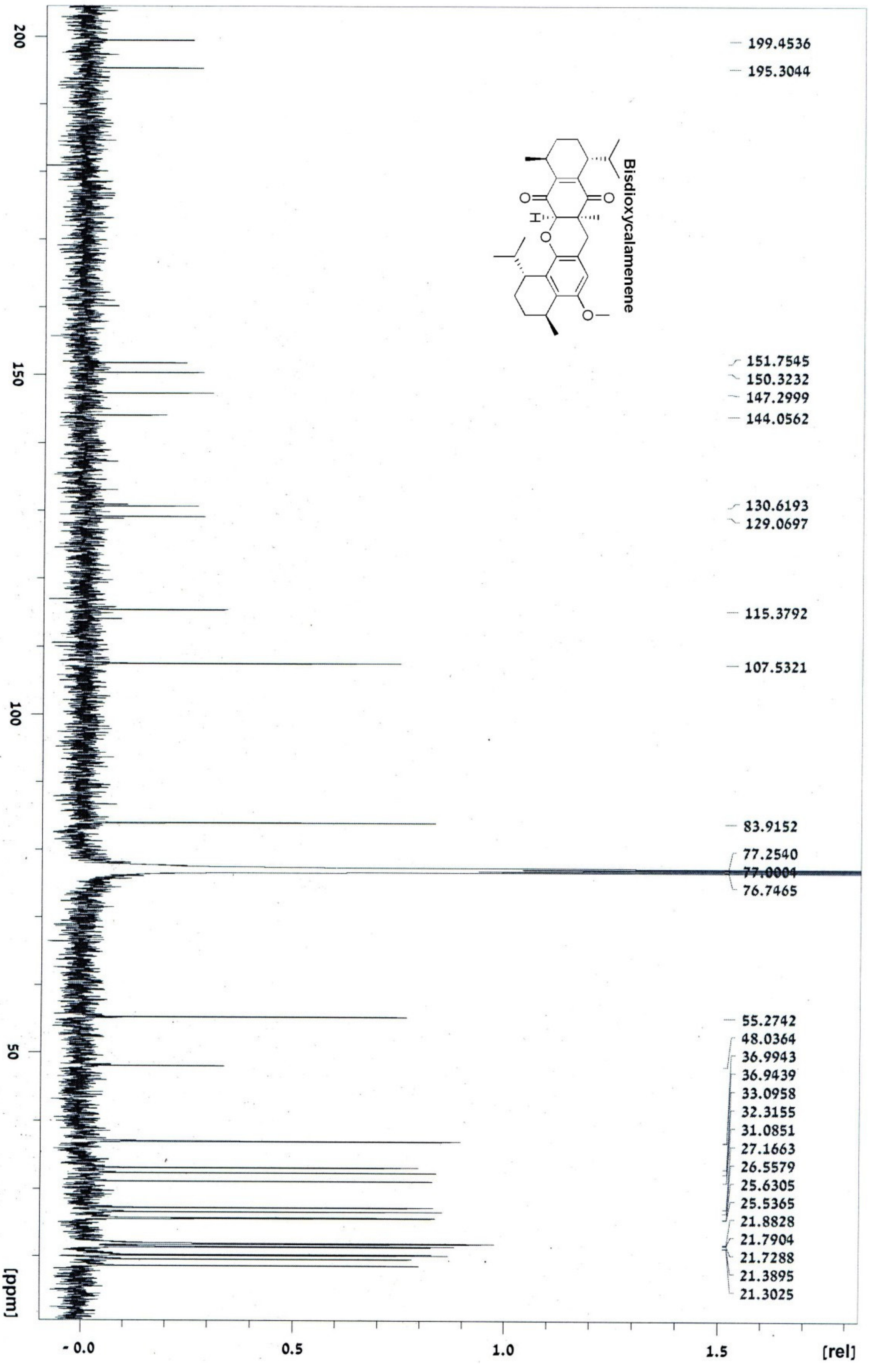

Figure S2. ^{13}C NMR of Bisdioxycalamenene (**1**) in $CDCl_3$.

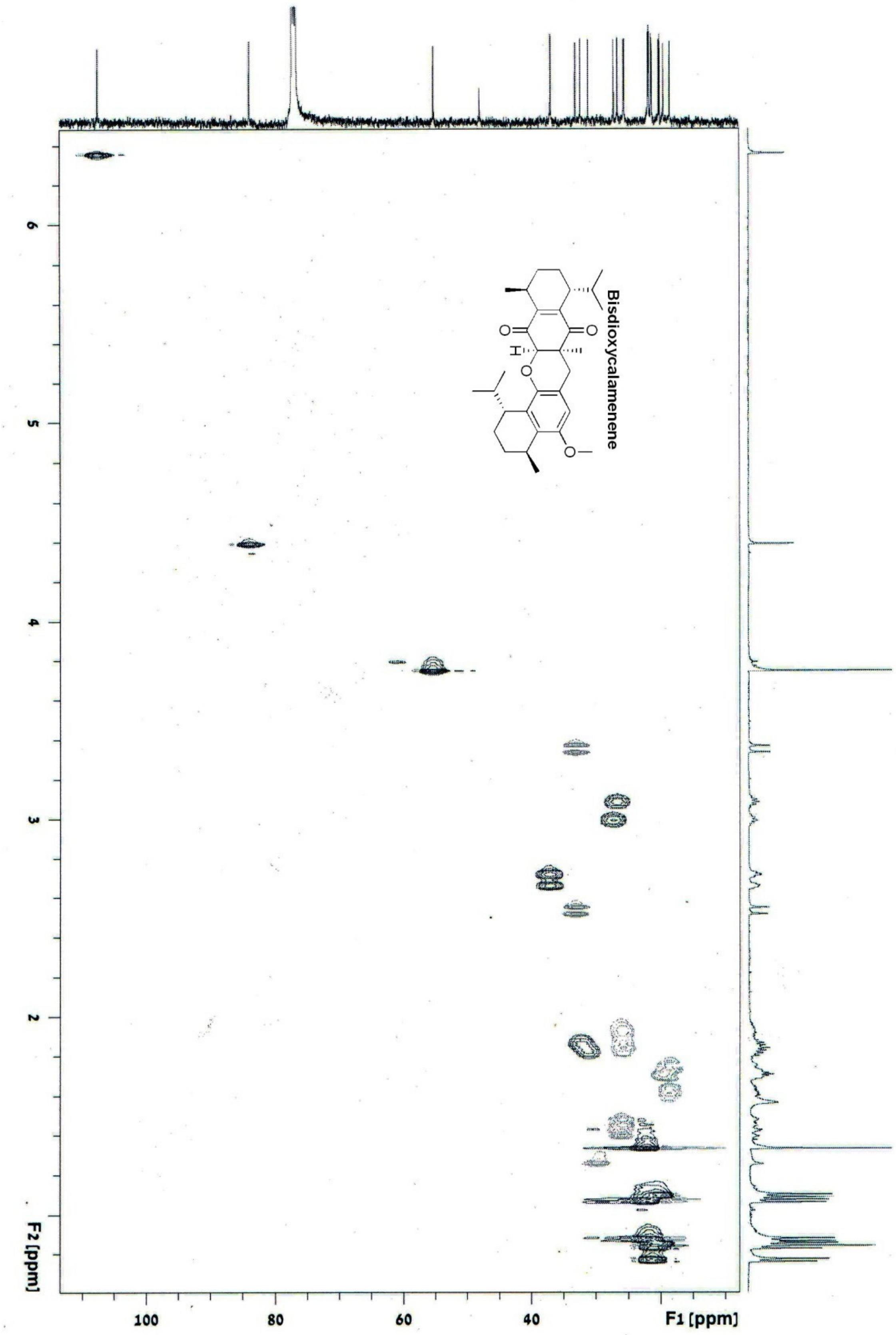

Figure S3. HSQC Spectrum of Bisdioxycalamenene (**1**) in $CDCl_3$.

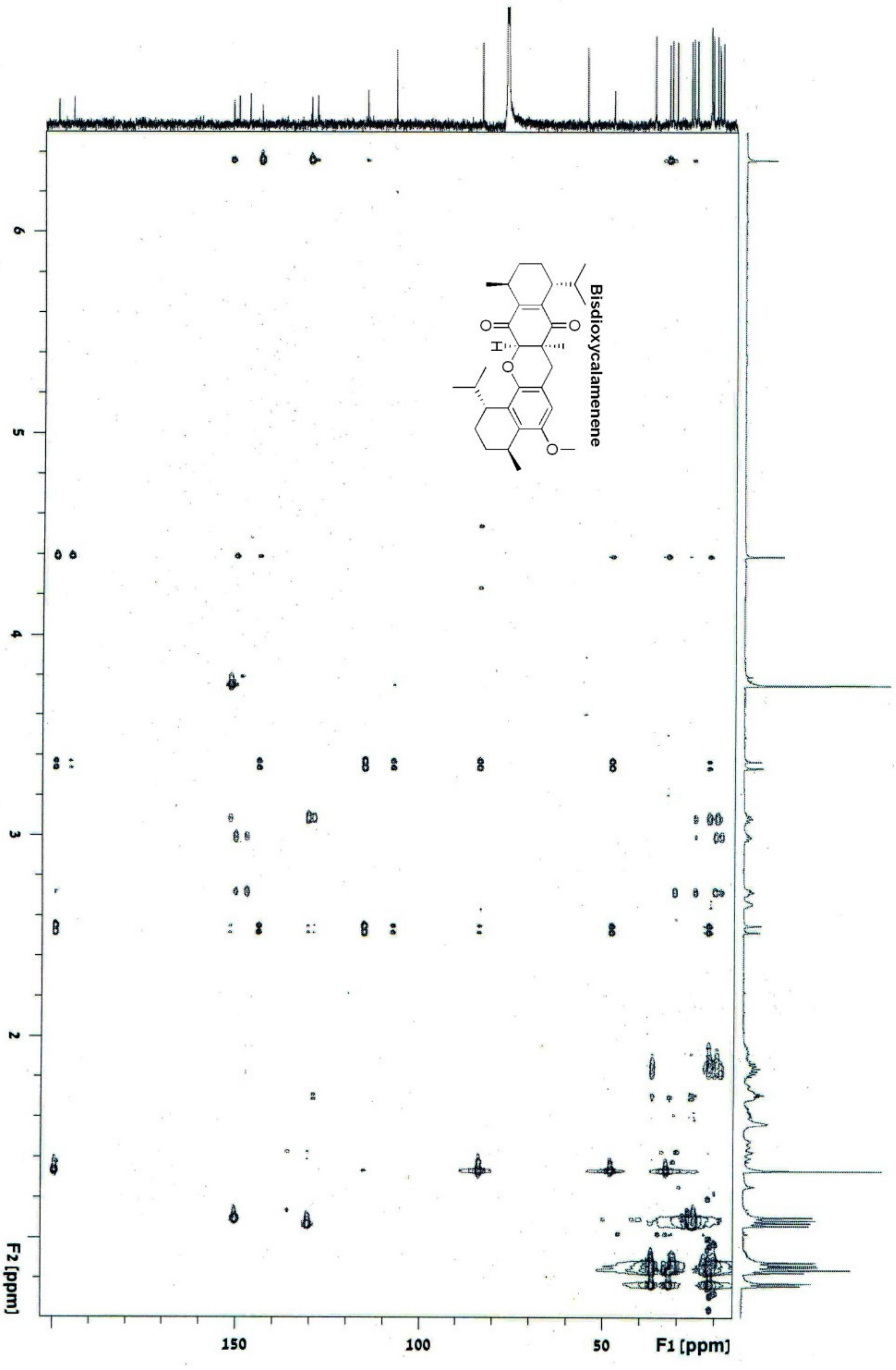

Figure S4. HMBC Spectrum of Bisdioxycalamenene (**1**) in $CDCl_3$.

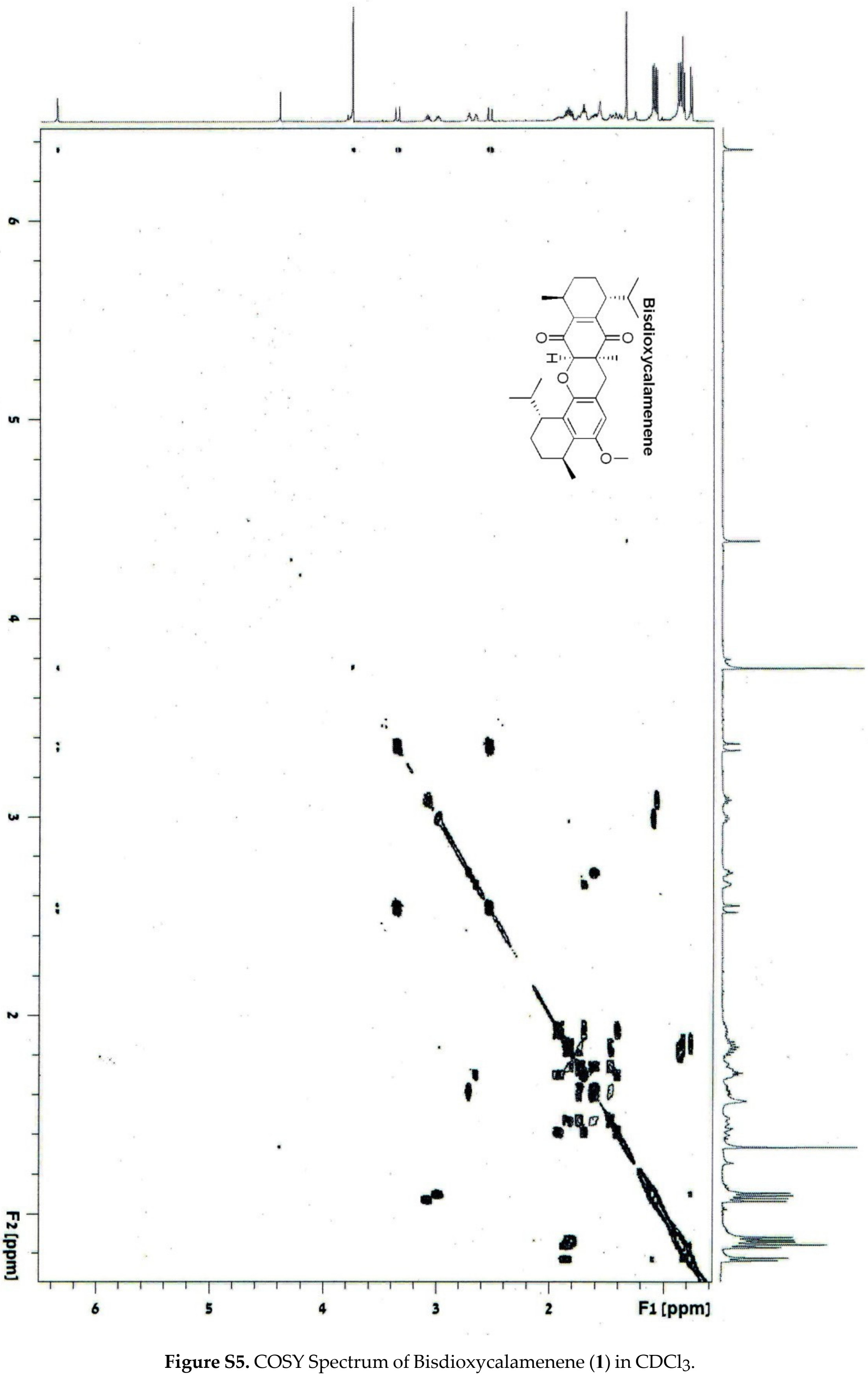

Figure S5. COSY Spectrum of Bisdioxycalamenene (**1**) in $CDCl_3$.

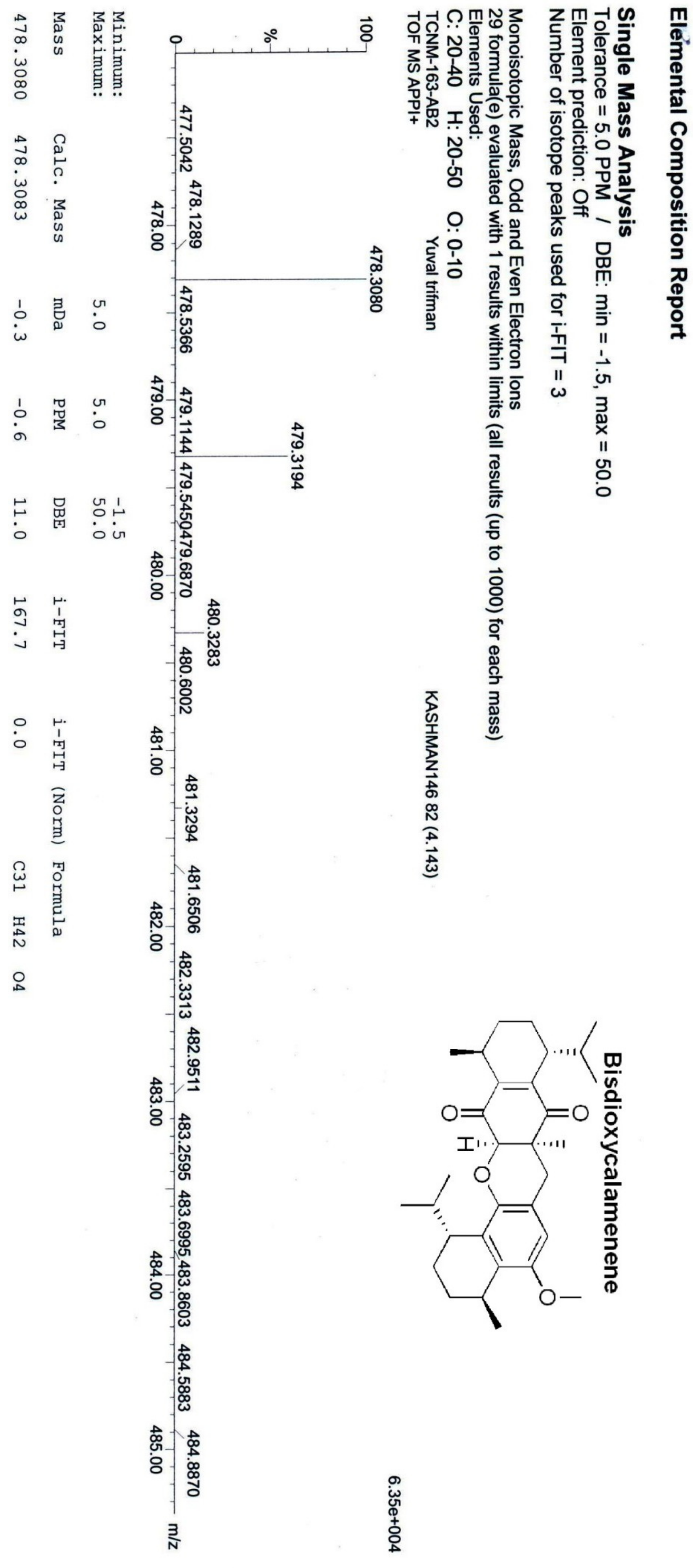

Figure S6. HRMS of Bisdioxycalamenene (**1**).

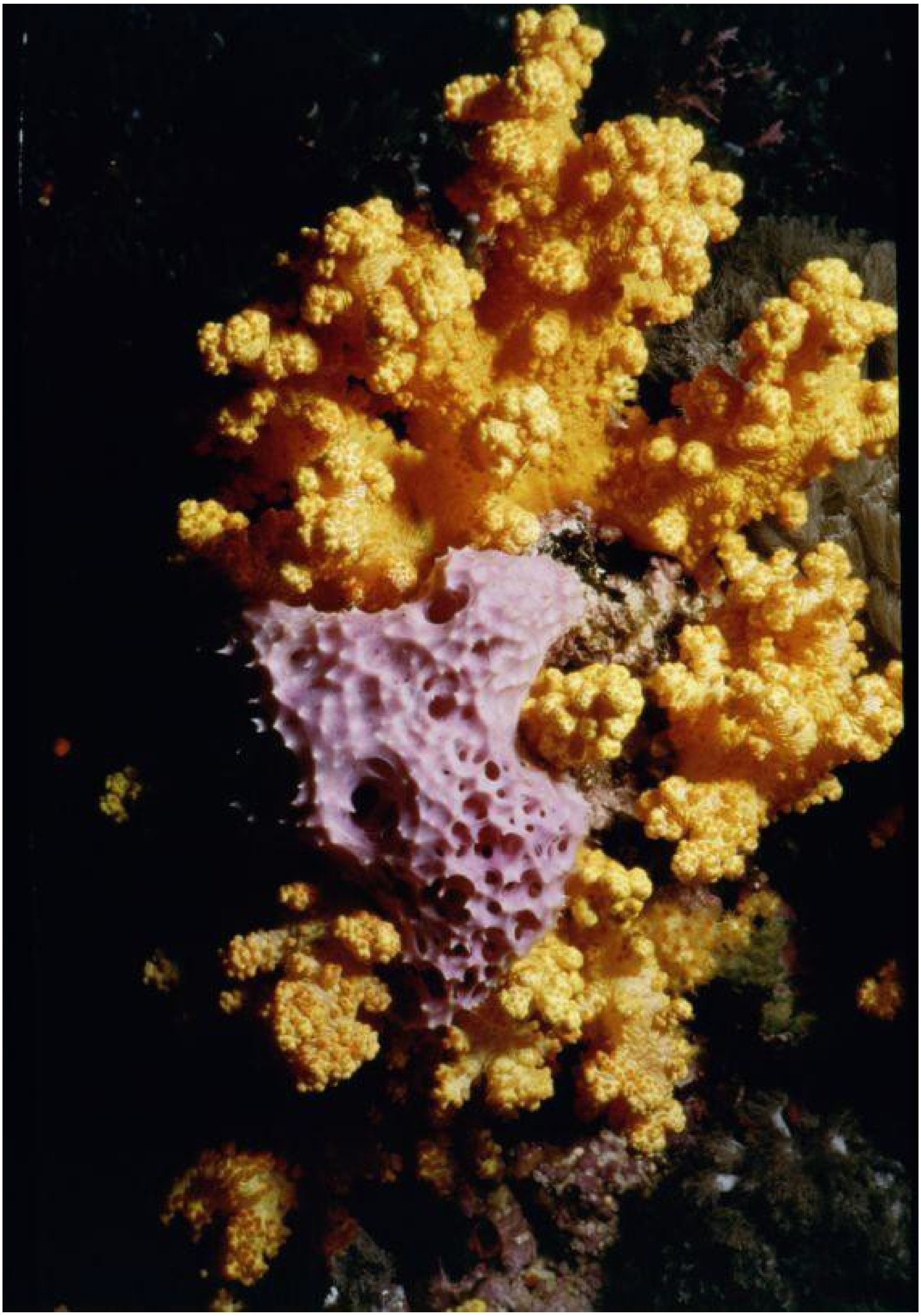

Figure S7. Image of Rhytisma *fulvum fulvum*. The soft coral (**yellow**) depicted on a sponge.
